# Conservative Management of Dental Erosion in Adolescents with Medical Conditions

**DOI:** 10.1155/2018/3230983

**Published:** 2018-12-16

**Authors:** Hikmah Mohd Nor, Nor Asilah Harun

**Affiliations:** ^1^Prosthodontics Department, Kulliyyah of Dentistry, International Islamic University Malaysia, Kuantan Campus, Bandar Indera Mahkota, 25200 Kuantan, Pahang, Malaysia; ^2^Paediatric Dentistry and Dental Public Health Department, Kulliyyah of Dentistry, International Islamic University Malaysia, Kuantan Campus, Bandar Indera Mahkota, 25200 Kuantan, Pahang, Malaysia

## Abstract

The prevalence of dental erosion among children and adolescents is trending higher in recent decades and is becoming a major concern. Dental erosion can be caused by either extrinsic or intrinsic acids or both. One of the established aetiological factors for dental erosion by intrinsic acid is the gastrooesophageal reflux disease. The degree of dental erosions may be influenced by any medical conditions that cause a reduction in salivary flow such as the salivary gland excision, autoimmune disease, radiation to the head and neck regions, and medications. If left untreated, the dental erosion can cause dentine hypersensitivity, loss of occlusal vertical height, and aesthetic problems. For effective management of dental erosion, the aetiology of each case must be determined, and its detection at an early stage is of prime importance. This case report illustrates the conservative management of dental erosion in two adolescent patients presented with their medical conditions and behaviour issues. The aim of the treatments was to preserve the vitality of the affected teeth. The treatments were successfully completed using a conservative approach, with the patients' medical conditions taken into consideration.

## 1. Introduction

Dental erosion is the irreversible loss of the dental hard tissue due to acid dissolution from either the extrinsic (e.g., dietary) or intrinsic (e.g., gastric) acids but not involving the bacterial plaque acid. Dental erosion is also not directly associated with mechanical or traumatic factors or with dental caries [1]. Caries, on the other hand, is the localized destruction of susceptible dental hard tissue caused by acidic by-products from bacterial fermentation of the dietary carbohydrates [2]. Nevertheless, the chemical process of dental erosion is similar to that of caries. Demineralization of the dental enamel will occur once the oral environmental pH reaches the critical threshold of 5.5 for both erosion and caries development [3, 4].

Dental erosion prevalence seems to be trending higher in recent decades and has become a concern especially when it happens in children and adolescent [5]. There are many indices used for the clinical evaluation of erosion. Therefore, it is difficult to accurately assess the prevalence of dental erosion from published epidemiological studies [6–8]. The prevalence of dental erosion has a great range, which can be explained by age, country, and different evaluation indices. In Malaysia, Milosevic and Lo [9] reported a prevalence of 95% and 41% of moderate and severe tooth wear, respectively, for the age group of 14–77 years, while Saerah et al. [6] reported a 100% prevalence of general tooth wear among 16-year-old secondary school children. The most recent study by Ab Halim et al. [10] reported that 99.8% out of the 598 16-year-old adolescents studied had at least one lesion with general tooth wear.

The aetiology of dental erosion can be from extrinsic and intrinsic origins. The extrinsic source of acids in the mouth is from the environment, diet, medications, and lifestyle. Meanwhile, the intrinsic source is from the backflow of gastric contents which is the hydrochloric acid in the mouth [11], either through vomiting or by regurgitation [12]. In children, dental erosion is primarily caused by excessive consumption of erosive food and drinks. It is found that soft drink has been associated with about 2.4-fold risk of dental erosion [13]. Common causes for the presence of the gastric acid in the oral cavity include gastroesophageal reflux disease (GORD), eating disorders, chronic vomiting, and persistent regurgitation and rumination [1]. In addition, the medical problems that cause a reduction in salivary flow include salivary gland excision, autoimmune disease, for example, Sjogren's syndrome, radiation to the head and neck region, and medications which can affect the degree of dental erosions [14].

Dental erosion has typical characteristics in terms of location, appearance, and morphology. The most frequently affected areas are the palatal surface of the maxillary incisors and the occlusal surface of the mandibular first molars in adolescents [15–17].

Early signs of erosion appear as smooth and flat facets on the facial or palatal surfaces and shallow and localized dimpling on the occlusal surfaces. This will eventually progress to deep cupping lesions with exposed dentin and loss of occlusal morphology without any intervention [15]. Consequently, this will cause dentine hypersensitivity, loss of occlusal vertical height, and aesthetic problems. For effective management of dental erosion, the aetiology of each case has to be fully understood, and it is of prime importance to recognize the problem at an early stage.

The following case studies illustrate the management of dental erosion cases of two adolescent patients with medical problems and behaviour issues. The treatment objectives were achieved by adopting conservative approaches, taking into considerations the medical conditions presented.

## 2. Case Studies

### 2.1. Case 1

A 10-year-old Caucasian male was referred by a general dental practitioner (GDP) for the management of dental caries and tooth surface loss. He had no complaint, and his dental problem was asymptomatic. He was diagnosed with infantile choriocarcinoma at the age of three weeks old. He had undergone chemotherapy for nine months and multiple surgical operations under general anaesthesia (GA) to remove the tumors and is currently in remission.

The patient also had kidney and gastric reflux diseases since he was 6 years old. Initially, he was treated with proton pump inhibitors (PPIs), but it failed to control the symptoms despite being given large doses of PPI. Eventually, at the age 8 years old, he underwent an antireflux surgery (fundoplication), wrapping the distal oesophagus with the uppermost part of his stomach. The surgery was successful in reducing his symptoms, and he was required to take 10 ml omeprazole every night. Unfortunately, on his 12^th^ visit to the dental clinic, the sutures of the gastric reflux surgery had ruptured because of prolonged vomiting during an episode of gastrointestinal infection, and a second surgery was planned in the future. The patient also had mild asthma, managed with salbutamol inhaler, and was known to have had an allergic reaction to vancomycin, Calpol®, and numerous “E” numbers food contents.

Diet history revealed diluted juice, isotonic drink, and flavoured water intake daily, and the patient frequently snacked on biscuits, chocolate, and sweets.

**Figure 1:**
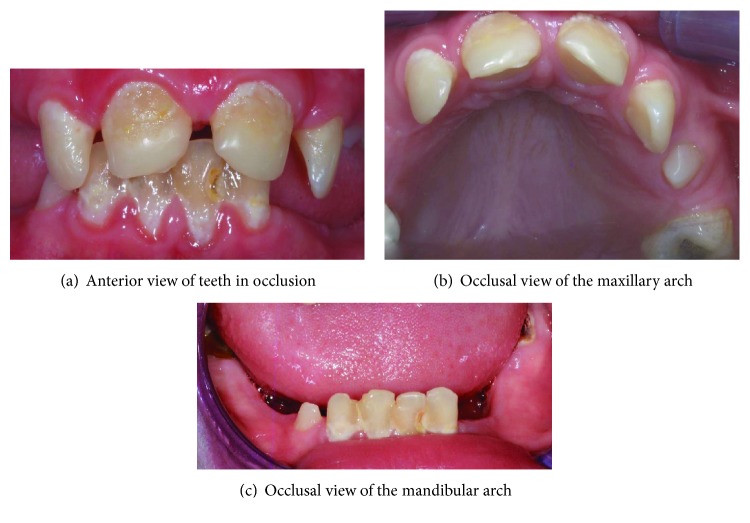
Pretreatment appearance of the dentition.

The clinical examination showed that most of his teeth were affected with caries (Figure 1). His oral hygiene was poor, and the lower left central incisor was nonvital. There were signs of erosion on the upper central incisors affecting the mesial and palatal surfaces.

**Figure 2:**
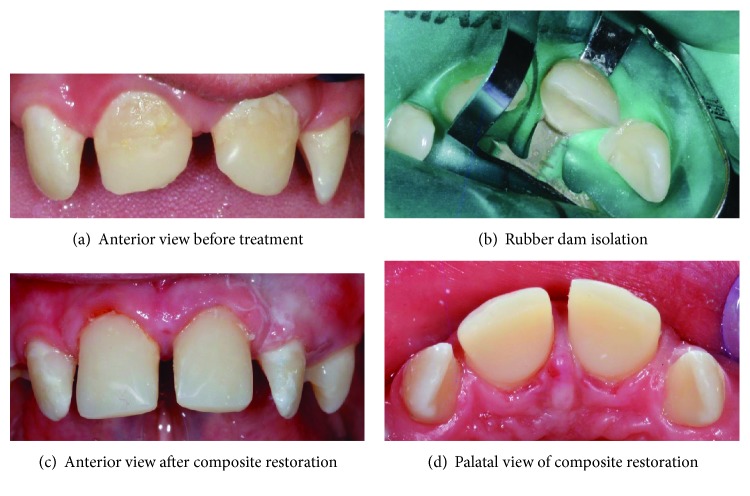
Composite restoration on 12 and composite built-up on tooth 11 and 22.

**Figure 3:**
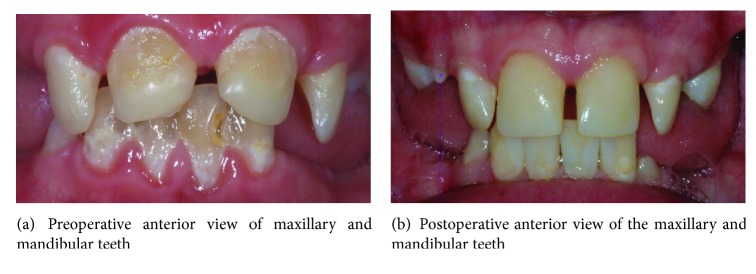
Comparison between the preoperative and postoperative anterior views.

A full mouth rehabilitation was carried out, which consisted of a root canal treatment on the lower left central incisor, composite restorations on all the incisors, and extractions of all the first permanent molars. The patient's dietary intake and oral hygiene were regularly monitored. Since the patient's gastric reflux problem was not under control due to the ruptured sutures of the previous fundoplication surgery, it was decided that the composite build-up treatment on the teeth affected by erosions should proceed. The upper incisor teeth were restored using microfilled composites resins (Durafill® VS) using the celluloid crown forms (Frasaco®) (Figure 2). A topical fluoride varnish application every 3-4 months was included in the treatment plan. In order to control the plaque formation, the patient was advised to brush his teeth using high fluoride toothpaste (more than 1350–1500 ppm fluoride) twice daily and to use a fluoride mouth rinse (FluoriGard® 0.05%NaF) daily at a different time apart from brushing. The patient was very cooperative throughout the treatment except during the first two visits whereby he required acclimatization and assurance. The patient was satisfied with the result of the treatment. The comparison between the preoperative and postoperative views of patient's dentition is shown in Figure 3.

### 2.2. Case 2

A 13-year-old Caucasian female was referred by a general dental practitioner (GDP) for the management of noncarious tooth surface loss of permanent maxillary anterior teeth. The patient did not complain of any pain or hypersensitivity and was asymptomatic.

His medical history revealed that the patient was diagnosed with hypoglycaemia when she was two years old. This rare condition, which was not related to diabetes, had caused her blood sugar to become low. She was on a daily carbohydrate supplement (Maxijul**®**) and consumed HypoStop**®** (concentrated glucose gel) whenever she had a hypoglycaemic attack. In addition, she had severe asthma that was managed with two puffs of salbutamol and steroid inhaler, twice daily, with a history of hospitalisation. The patient also had a migraine attack every other day, usually followed by vomiting and nausea since she was eight years old. She was under the treatment of a consultant paediatric neurologist and was managed with sumatriptan and Migraleve for her migraine and domperidone to prevent vomiting. She was also known to have an allergy to peanuts. She was a regular dental attendee and had experienced dental treatment and tooth extraction under local analgesia.

Her dietary history showed that the patient consumed excessive quantities of acidic beverages: Coca-Cola**®**, Irn-Bru**®**, and diluted fruit juice. The diluted juice was mixed with Maxijul**®** and was taken to bed at night, and the mixed juice was sipped throughout the night.

**Figure 4:**
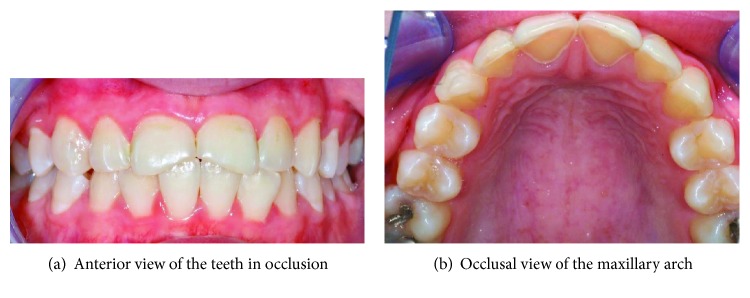
Pretreatment appearance of the dentition.

Clinical examination revealed generalised dental erosion that had severely affected the palatal surfaces of all the maxillary incisors and caused a fracture of the mesial surface of both the maxillary central incisors (Figure 4). Several restorations were present on the posterior teeth.

The patient was very anxious during the first visit to the clinic. The clinical and radiographic examinations were completed after the patient was successfully coaxed and reassured. The impressions of the maxillary and mandibular teeth were made during the second visit. Acclimatization, reassurance, tell-show-do, and distraction were accomplished throughout the procedure because the patient had a pronounced gag reflex. Alginate impression materials of different tastes were used to encourage the patient's cooperation.

**Figure 5:**
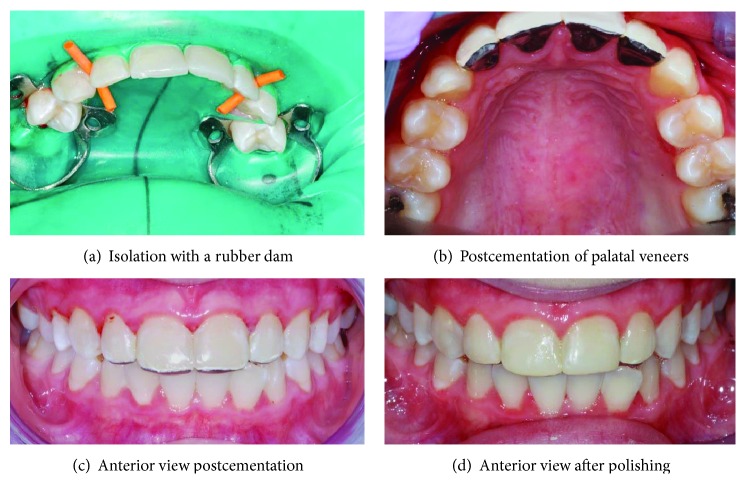
Cementation of the palatal veneers.

At subsequent visits, the patient was cooperative and happy to continue with the treatment. Consent was obtained for the central maxillary incisors to be restored under rubber dam isolation and local anaesthesia. The teeth were restored with microfilled composite resins (Durafill® VS) shade A1 with the used of celluloid crown forms (Frasaco®). Both the patient and her mother were happy with the results. Following that, the patient became more confident and displayed less anxiety. She was happy to proceed with the impression making procedures for the construction of the nickel-chromium palatal veneers for all the maxillary incisors, using heavy body polyvinyl siloxane impression material and light body polyvinyl siloxane impression material. The cementation of the nickel-chromium palatal veneers was done during the following visit using Panavia™ F2.0 resin cement (Figure 5). The aesthetic and function were found to be satisfactory. The patient's diet and oral hygiene were regularly monitored posttreatment, and the applications of topical fluoride varnish were done every 3-4 months. She was prescribed a high fluoride toothpaste (2800 pm fluoride) to be used twice daily and was advised to use a fluoride mouth rinse (FluoriGard® 0.05% NaF) daily at a different time apart from the time of brushing her teeth.

## 3. Discussion

Infantile choriocarcinoma, suffered by case 1 patient, is a malignant growth of the trophoblastic cells characterized by the secretion of human chorionic gonadotropin (hCG). It is a very rare tumor which occurs approximately once in every 40,000 pregnancies [18]. It is highly malignant in nature, and prior to the advert of chemotherapy, its prognosis was poor [19]. Multiagent chemotherapy combined with surgical resection has proven to be successful for infantile choriocarcinoma [20].

As a result of a series of chemotherapy for a period of nine months from the age of three weeks old, the patient's erupting mandibular canines are microdontia. Studies have shown the possibility of having microdontia teeth in the permanent dentition following chemotherapy below the age of 3.5 years old [21]. Chemotherapy may result in an increased incidence of dental developmental disturbances such as the failure of teeth to develop, hypodontia, enamel hypoplasia, enamel defect, and discolouration [22–24]. It was found that all of the patient's first permanent molars were carious and it was not clear if the enamel defect was the predisposing factor.

Cases 1 and 2 both experienced tooth surface loss due to erosion. The aetiology of erosion has to be identified to ensure the appropriate management can be carried out. Based on the patients' history, the possible aetiological factors for the tooth surface loss were the intrinsic acid source from vomiting and gastrooesophageal reflux disease (GORD) and also the extrinsic source from acidic drink such as diluted juice and carbonated drink that they consumed. Both of them suffered from asthma and the disease was managed using the beta-2 agonist inhaler. The daily use of salbutamol can cause a reduction in the buffering capacity and salivary flow rate. Therefore, it can potentially increase the susceptibility of teeth to acid attack. This has been reported to be a predisposing factor for erosion and caries incidence [25, 26]. However, conversely, a study done by Dugmore and Rock [27] reported a lack of association between asthma and tooth erosion.

The aims of the treatment for tooth surface loss are to resolve sensitivity if present, to restore the missing tooth tissue, for functional and aesthetic purposes, to prevent further tooth surface loss, and to maintain occlusion.

In the case 1 patient, the maxillary incisors were restored by direct composite build-ups. As the patient was in a mixed dentition, the resin-based restoration was decided to be the treatment of choice [28]. This is a conservative treatment with no tooth preparation and it has a good long-term survival rate of up to 89% for 56 months [29]. A direct composite resin restoration can be done in a single visit. It is also easy to maintain for it can be added to and repaired. The composite resin restorations that were used to treat the localized anterior tooth wear had a good short- to medium-term survival and the median survival rate of 4 years 9 months [30]. The restorations may require regular maintenance due to chipping, debonding, and/or discolouration [31] which can easily be done during a review visit.

In the case 2 patient, the erosion at the mesial surfaces of the maxillary central incisors was restored with direct composite resins at the buccal surfaces to obtain an aesthetic result, and the erosion on the palatal surfaces of the teeth was restored with nickel-chromium palatal veneers. As there were signs of erosion in combination with some degree of attrition seen in this patient, the aim of the treatment was to prevent further palatal tooth surface loss and reduce the risk of incisal edge fracture. The resin-bonded nickel-chromium palatal veneers were considered to be suitable in managing this case [1, 32]. By covering the palatal surfaces including the incisal edges with alloy veneers, the resistance of the shearing loads is expected to be increased, and it will minimise the wear of opposing natural dentition [28, 33]. It was found that for the palatal veneers, nickel-chromium had the highest tensile strength compared with gold alloy (type III), composite resin restoration and porcelain, low wear rate against the opposing enamel when compared with the porcelain, and it has good corrosion resistance [32, 34]. Adversely, nickel-chromium can be difficult to polish [34], and it is not aesthetically appealing due to the metallic appearance. The good cooperation displayed by the patient throughout the treatment resulted in successful cementation of the nickel-chromium palatal veneers.

Although there were studies that suggested hybrid composite resins as the material of choice for the management of the tooth surface loss [35–37], for both cases, the microfilled composite resins were chosen because of the excellent aesthetics due to the lower filler content and high polishability [38]. A study also found that the Durafill® VS microfilled composite resins had the lowest polymerisation shrinkage compared with the traditional hybrid composites [39].

A localized increase in the occlusal vertical dimension (OVD) was carried out in these two cases using the “Dahl concept” to provide space for the restorations without the removal of a sound tooth tissue [31]. The nickel-chromium veneers and composite restorations were placed on the palatal of the incisors, acting as a fixed Dahl appliance which created an increase in the OVD. A posterior occlusion was found to be reestablished after a month, and both patients tolerated it well with the increase in the OVD. The “Dahl concept” involved both the compensatory eruption of the posterior teeth and intrusion of the anterior teeth [40]. This technique was found to be safe and able to avoid destructive restorative procedures on the teeth that had already been compromised [31].

Dietary advice was given in accordance with the Scottish Intercollegiate Guidelines Network (SIGN 47) [41] to both patients and their parents which included advice on restricting diluted juice and carbonated drink consumption to mealtimes only, eating healthy snacks and drinking milk or water in-between meals. The patients were advised to brush their teeth using a high fluoride toothpaste (2800 pm fluoride). In addition, they were advised not to drink or eat after brushing their teeth at night. The daily use of fluoride mouth rinse (FluoriGard® 0.05%NaF) at a different time apart from the time of teeth brushing helped to maximise fluoride exposure and, thus, encourage the remineralisation process of the incipient enamel caries lesions. The fluoride therapy was further enhanced with professionally applied fluoride varnish (Duraphat®) with 22,600 ppm fluoride every 3 to 4 months [42].

Initially, both patients were very anxious about having dental treatments, but after a few appointments, they appeared more confident in the dental setting and were coping very well with the dental treatments under local anaesthesia. Behaviour guidance techniques were adopted in the management of the patients. It was a comprehensive, continuous process to develop the relationship between the patient and doctor which eventually builds trust and eases the patients' fear and anxiety [43]. The methods used such as acclimatization, reassurance, tell-show-do, and distraction helped to instil confidence and positive dental attitude in both patients.

## 4. Conclusion

Comprehensive dental management is vital in providing dental treatment to patients with special health care needs. The prevention of dental diseases from an early age is crucial to ensure that the patients' teeth condition, masticatory function, and aesthetic will not be affected, with the hope that their teeth will last a lifetime. For cases presented with dental erosions at the palatal surface of maxillary incisors, placing cast-metal palatal veneers or composite resin restorations with a localized increase in the OVD is recommended. These techniques provide options to restore the eroded teeth without the sound tooth tissue being removed. Ensuing the restorative management, patients will be required to be closely monitored and preventive reinforcements need to be frequently established. The patients need to understand their part in preventing dental disease by adopting healthy eating habits and maintaining their oral hygiene.

## References

[1] O'Sullivan E., Milosevic A., British Society of Paediatric Dentistry (2008). UK National Clinical Guidelines in Paediatric Dentistry: diagnosis, prevention and management of dental erosion. *International Journal of Paediatric Dentistry*.

[2] Selwitz R. H., Ismail A. I., Pitts N. B. (2007). Dental caries. *The Lancet*.

[3] Moynihan P., Petersen P. E. (2004). Diet, nutrition and the prevention of dental diseases. *Public Health Nutrition*.

[4] García-Godoy F., Hicks M. J. (2008). Maintaining the integrity of the enamel surface. *Journal of the American Dental Association*.

[5] Nunn J. H., Gordon P. H., Morris A. J., Pine C. M., Walker A. (2003). Dental erosion–changing prevalence? A review of British national childrens’ surveys. *International Journal of Paediatric Dentistry*.

[6] Saerah N. B., Ismail N. N., Naing L., Ismail A. R. (2006). Prevalence of tooth wear among 16-year-old secondary school children in Kota Bharu Kelantan. *Archives of Orofacial Sciences*.

[7] Bardsley P. F. (2008). The evolution of tooth wear indices. *Clinical Oral Investigations*.

[8] Lussi A., Schlueter N., Rakhmatullina E., Ganss C. (2011). Dental erosion - an overview with emphasis on chemical and histopathological aspects. *Caries Research*.

[9] Milosevic A., Lo M. S. (1996). Tooth wear in three ethnic groups in Sabah (northern Borneo). *International Dental Journal*.

[10] Ab Halim N., Esa R., Chew H. P. (2018). General and erosive tooth wear of 16-year-old adolescents in Kuantan, Malaysia: prevalence and association with dental caries. *BMC Oral Health*.

[11] Corica A., Caprioglio A. (2014). Meta-analysis of the prevalence of tooth wear in primary dentition. *European Journal of Paediatric Dentistry*.

[12] Moazzez R., Bartlett D. (2014). Intrinsic causes of erosion. *Monographs in Oral Science*.

[13] Li H., Zou Y., Ding G. (2012). Dietary factors associated with dental erosion: a meta-analysis. *PLoS One*.

[14] Paryag A., Rafeek R. (2014). Dental erosion and medical conditions: an overview of aetiology, Diagnosis and Management. *West Indian Medical Journal*.

[15] Ren Y.-F. (2013). Dental erosion: etiology, diagnosis and prevention. *RDH*.

[16] Johansson A.-K., Omar R., Carlsson G. E., Johansson A. (2012). Dental erosion and its growing importance in clinical practice: from past to present. *International Journal of Dentistry*.

[17] El Aidi H., Bronkhorst E. M., Truin G. J. (2008). A longitudinal study of tooth erosion in adolescents. *Journal of Dental Research*.

[18] Yoon J. M., Burns R. C., Malogolowkin M. H., Mascarenhas L. (2007). Treatment of infantile choriocarcinoma of the liver. *Pediatric Blood & Cancer*.

[19] Dumesnil C., Gatbois E., Leverger G. (2005). Le choriocarcinome infantile: une tumeur exceptionnelle et curable. *Archives de Pédiatrie*.

[20] Hanson D., Walter A. W., Dunn S., Rittenhouse D. W., Griffin G. (2011). Infantile choriocarcinoma in a neonate with massive liver involvement cured with chemotherapy and liver transplant. *Journal of Pediatric Hematology/Oncology*.

[21] Hutton A., Bradwell M., English M., Chapple I. (2010). The oral health needs of children after treatment for a solid tumour or lymphoma. *International Journal of Paediatric Dentistry*.

[22] Welbury R. R., Craft A. W., Murray J. J., Kernahan J. (1984). Dental health of survivors of malignant disease. *Archives of Disease in Childhood*.

[23] Maguire A., Craft A. W., Evans R. G. B. (1987). The long-term effects of treatment on the dental condition of children surviving malignant disease. *Cancer*.

[24] Oguz A., Cetiner S., Karadeniz C., Alpaslan G., Alpaslan C., Pinarli G. (2004). Long-term effects of chemotherapy on orodental structures in children with non-Hodgkin’s lymphoma. *European Journal of Oral Sciences*.

[25] Al-Dlaigan Y. H., Shaw L., Smith A. J. (2002). Is there a relationship between asthma and dental erosion? A case control study. *International Journal of Paediatric Dentistry*.

[26] Thomas M., Parolia A., Kundabala M., Vikram M. (2010). Asthma and oral health: a review. *Australian Dental Journal*.

[27] Dugmore C. R., Rock W. P. (2003). Asthma and tooth erosion. Is there an association?. *International Journal of Paediatric Dentistry*.

[28] Johansson A., Johansson A. K., Omar R., Carlsson G. E. (2008). Rehabilitation of the worn dentition. *Journal of Oral Rehabilitation*.

[29] Nohl F. S., King P. A., Harley K. E., Ibbetson R. J. (1997). Retrospective survey of resin-retained cast-metal palatal veneers for the treatment of anterior palatal tooth wear. *Quintessence International*.

[30] Redman C. D. J., Hemmings K. W., Good J. A. (2003). The survival and clinical performance of resin–based composite restorations used to treat localised anterior tooth wear. *British Dental Journal*.

[31] Poyser N. J., Porter R. W. J., Briggs P. F. A., Chana H. S., Kelleher M. G. D. (2005). The Dahl concept: past, present and future. *British Dental Journal*.

[32] Chu F. C. S., Siu A. S. C., Newsome P. R. H., Chow T. W., Smales R. J. (2002). Restorative management of the worn dentition: 2. Localized anterior toothwear. *Dental Update*.

[33] Khan F., Young W. G. (2011). *Toothwear: The ABC of the Worn Dentition: Khan/Toothwear: The ABC of the Worn Dentition*.

[34] McCabe J. F., Walls A. (2008). *Applied Dental Materials*.

[35] Milosevic A. (2018). Clinical guidance and an evidence-based approach for restoration of worn dentition by direct composite resin. *British Dental Journal*.

[36] Milosevic A., Burnside G. (2016). The survival of direct composite restorations in the management of severe tooth wear including attrition and erosion: a prospective 8-year study. *Journal of Dentistry*.

[37] Hamburger J. T., Opdam N. J., Bronkhorst E. M., Kreulen C. M., Roeters J. J., Huysmans M. C. (2011). Clinical performance of direct composite restorations for treatment of severe tooth wear. *The Journal of Adhesive Dentistry*.

[38] Von Fraunhofer J. A. (2013). *Dental Materials at a Glance*.

[39] Blackham J. T., Vandewalle K. S., Lien W. (2009). Properties of hybrid resin composite systems containing prepolymerized filler particles. *Operative Dentistry*.

[40] Loomans B., Opdam N., Attin T. (2017). Severe tooth wear: European consensus statement on management guidelines. *The Journal of Adhesive Dentistry*.

[41] Scottish Intercollegiate Guidelines Network (SIGN) (2000). Preventing dental caries in children at high caries risk. Targeted prevention of dental caries in the permanent teeth of 6-16 year olds presenting for dental care (SIGN 47).

[42] NHS (2009). *Deliverying Better Oral Health: An Evidence-Based Toolkit for Prevention*.

[43] AAPD (2011). Guideline on behavior guidance for the pediatric dental patient. *Reference Manual*.

